# Supervised redundant feature detection for tumor classification

**DOI:** 10.1186/1755-8794-7-S2-S5

**Published:** 2014-10-22

**Authors:** Xue-Qiang Zeng, Guo-Zheng Li

**Affiliations:** 1 Computer Center, Nanchang University, 999 Xuefu Road, Nanchang, 330029, China; 2 Department of Control Science & Engineering, Tongji University, 4800 Cao An Road, Shanghai, 201804, China; 3 The Key Laboratory of Embedded System & Service Computing, Tongji University, 4800 Cao An Road, Shanghai, 201804, China

**Keywords:** Feature selection, Redundant feature, Microarray data, Tumor classification

## Abstract

**Background:**

As a high dimensional problem, analysis of microarray data sets is a challenging task, where many weakly relevant or redundant features affect overall performance of classifiers.

**Methods:**

The previous works used redundant feature detection methods to select discriminative compact gene set, which only considered the relationship among features, not the redundancy of classification ability among features. This study propose a novel algorithm named RESI (Redundant fEature Selection depending on Instance), which considers label information in the measure of feature subset redundancy.

**Results:**

Experimental results on benchmark data sets show that RESI performs better than the previous state-of-the-art algorithms on redundant feature selection methods like mRMR.

**Conclusions:**

We propose an effective supervised redundant feature detection method for tumor classification.

## Background

Rapid advances in gene expression microarray technology enable simultaneous measurement of the expression levels for thousands or tens of thousands of genes in a single experiment. Analysis of microarray data presents unprecedented opportunities and challenges for data mining in areas such as gene clustering, class discovery, and tumor classification [1]. A typical data set may contain thousands of genes but only a small number of samples (often less than a hundred). The number of samples is likely to remain small at least for the near future due to the expense of collecting microarray samples [2]. The nature of relatively high dimensionality but small sample size in microarray data causes the known problem of "curse of dimensionality". Therefore, selecting a small number of discriminative ones from thousands of genes is essential for successful sample classification [3-5].

Feature selection, a process of choosing a subset of features from the original, is frequently used as a preprocessing technique in data mining. It has been proved effective in reducing dimensionality, improving mining efficiency, increasing mining accuracy, and enhancing result comprehensibility [6-9]. The goal of the feature selection algorithm is to select the minimum set of features that are strongly related to the desired decision variable and have the least redundancy among them [10,11]. Existing efficient feature selection algorithms (feature ranking methods) usually assume feature independence, and assign a discriminative score to each feature. Because the interactions and correlations among features are not considered, these algorithms fail to remove redundant features completely.

Hall [12] pointed out that the prediction capability and the inter-correlation of feature subset are two important aspects in feature selection. However, the optimal feature selection requires an exponentially large search space (*O*(2*^m^*), where *m *is the number of features) [10]. In practise, researchers often resort to various approximations to determine the optimal feature subset. The prediction capability is usually estimated by the interrelation of individual feature with the target. For the issue of redundancy, many researchers attempt to explicitly address feature interactions by finding some low-order interactions, *i.e*. 2-way (one feature and the label) and 3-way (two features and the label) interactions.

In recent years, researchers have proposed many techniques to eliminate redundant features according to the above principle. Battiti proposed the Mutual Information based Feature Selection (MIFS) method [13], and then improved versions like MIFS-U [14] and mMIFS-U [15] were proposed. Hall proposed the Correlation-based Feature Selection (CFS) method [12], Ding and Peng proposed the minimum Redundancy-Maximum Relevance (mRMR) method [16], Bontempi and Meyer proposed a Causal filter selection method, called min-Interaction Max-Relevance (mIMR) [17], Fleuret used the Conditional Mutual Information Maximization (CMIM) to select feature [18]. These methods consider the problem of feature selection from different motivations and various solutions have been proposed. But the same point is that they are trying to find the optimal discriminative feature subset by considering to remove feature redundancy, where feature redundancy is computed by various pair-wise similarity measures, *i.e*. mutual information or conditional mutual information.

However, the used traditional pair-wise similarity metrics only consider the numerical values of given variables, but not the similarity of discriminative ability among them. Therefore, feature redundancy can not be measured correctly in terms of feature prediction ability by existing methods. How to measure feature (subset) redundancy is a fundamental problem, which should be reconsidered in the field of feature selection. In the application of classification, we consider a feature redundant only when its predictive power is redundant, not that its numerical value is similar with some selected features. Because two highly similar features are obviously not redundant to each other when the minor difference happen to be critical to the classification. In this paper, we proposed a novel method to measure feature (subset) redundancy by comparing features' predictive powers directly. Feature's prediction power is recorded by its instances' distribution explicitly, which includes clear-discerned instances and blur-discerned instances. Based on the predictive power distributions, a new feature redundancy metric is defined on the ground of comparing predictive powers. Furthermore, we proposed a novel algorithm named RESI (Redundant fEature Selection depending on Instance). Experiments on several benchmark microarray data sets demonstrate the outstanding performance of our proposed method.

## Methods

### Supervised redundant feature detection

Given a data set (*X *= [*X*_1_, *X*_2_, . . . , *X_m_*], *Y*) with *n *instances represented by *m *features (genes), where *X *and *Y *are instances matrix and class label respectively. The task of classification is to tag instances with a label in low probability of error. Theoretically, having more features implies more discriminative power in classification. However, many features are relevant to each other and they have no contribution to classification, except to degrade performance of classifiers [19]. Thus, it is necessary to remove irrelevant and redundant features as more as possible, without losing information greatly. Generally, the task of feature selection in classification issue is formally defined as the process of selecting the optimal feature subset *S *⊆ *X *to have high classification performance.

Thus, qualities of relevant and non-redundant features of the selected subset are two key points considered in feature selection methods. In existing methods, predictive power of a feature subset has often been divided into computation of the interrelation score of individual features with the label. Due to computational limitation in high dimensional feature space, redundancy degree of a feature subset is usually estimated by pair-wise similarity measures, *i.e*. mutual information or conditional mutual information of two given variables.

However, to measure the feature subset redundancy is a fundamental problem, which should be reconsidered in the field of feature selection. Without including learning model (*i.e*. wrapper methods), measuring the redundancy degree of a feature subset as a whole is impractical. So, approximately measuring individual pair-wise feature similarity is a reasonable way which has been adopted by various existing methods. But, the previous pair-wise similarity measures only consider the numerical values of given features, but not the discriminative ability among them. Therefore, feature redundancy is not measured correctly. For instance, two highly similar features are usually considered as redundant to each other by the previous pair-wise similarity measures. But this is not correct when the minor difference of the two features happens to be critical to the classification.

In the context of classification, it is reasonable to address the feature set redundancy from the view of predictive power. So, we define the pair-wise feature redundancy as follow.

**Theorem 1 **In the context of classification, a feature X_p _is redundant to a given feature X_q _if its predictive power to label Y has already been expressed by feature X_q_.

As argued in Theorem 1, the similarity should be measured by comparing the distribution of predictive power between two features. Thus, the predictive power needs to be measured in a comparable way. More concretely, we need to record those instances which are clear-discerned by the given feature and which are not.

**Theorem 2 **Given a feature X_p_, an instance X^u ^is clear-discerned if the majority of its k nearest neighbour instances on feature X_p _have the same class labels with that of X^u^.

The intuitive idea of Theorem 2 is inspired from the *k *Nearest Neighbor (*k*NN) classifier. We believe that Theorem 2 holds true no matter which classification model is applied finally. Based on the idea in Theorem 2, we define the neighborhood pUrity of instance *X^u ^*on a given feature *X_p _*as below,

(1) $$U\left(X_{p}^{u}|Y\right)=\frac{\sum_{X^{v}\in N_{k}\left(X_{p}^{u}\right)}\delta \left(Y^{v}==Y^{u}\right)}{k},$$

where *δ*(·) is the Kronecker delta function *i.e. δ*(·) = 1 when *Y^v ^*== *Y^u ^*is true, otherwise *δ*(·) = 0, *Y^u ^*is the target label of instance *X^u^*, $X_{p}^{u}$ is the value of instance *X^u ^*on feature *X_p_, N_k _*($X_{p}^{u}$) is the neighborhood of *X^u ^*defined by the *k *closest instances *X^v ^*on feature *X_p _*($X_{p}^{v}$ is close to $X_{p}^{u}$), *k *is a predefined parameters. Equation 1 measures how many instance labels are the same in the neighborhood of *X^u ^*via feature *X_p_*.

The value of $U\left(X_{p}^{u}|Y\right)$ varies from 0 to 1. High $U\left(X_{p}^{u}|Y\right)$ means the corresponding instance has more neighbors with the same class. An instance is defined as clear-discerned when its $U\left(X_{p}^{u}|Y\right)$ is higher than a given threshold *µ, i.e*. $U\left(X_{p}^{v}|Y\right)>\mu$, otherwise it is blur-discerned.

By Equation 1, each feature's predictive power is recorded as a distribution of instances, *i.e*. the clear-discerned instances and the blur-discerned instances. Then, redundancy of two given feature can be measured by comparing the corresponding instance distributions directly. We define the REdundancy Measure depending on Instances (REMI) of feature *X_q _*to feature *X_p _*under label *Y *as follows.

(2) $$REMI\left(X_{p};X_{q}|Y\right)=\frac{\sum_{u=1}^{n}\delta \left(U\left(X_{p}^{u}|Y\right)\leq \mu \;\;\&\;\;U\left(X_{q}^{u}|Y\right)\leq \mu \right)}{\sum_{u=1}^{n}\delta \left(U\left(X_{p}^{u}|Y\right)\leq \mu \right)}$$

The numerator of Equation 2 gives the count of instances which are blur-discerned on both feature *X_p _*and feature *X_q_*, the denominator is the blur-discerned instances which count on feature *X_p_*. The value of *REMI*(*X_p_; X_q_*|*Y*) varies from 0 to 1. It is 0, when all blur-discerned instances on feature *X_p _*are clear-discerned on feature *X_q_*, which means the predictive power of *X_q _*is completely complementary to that of *X_p _*and we consider their corresponding redundancy is zero. The value is 1 when no blur-discerned instance on feature *X_p _*is clear-discerned on feature *X_q_*, which means *X_q _*has no predictive power contribution to *X_p_*, i.e. *X_q _*is redundant to *X_p_*.

As to measure the predictive power of selected features, we use the metric of t-statistic. For binary classification, the definition of t-statistic on a feature *p *is given as:

(3) $$t\left(p|Y\right)=\frac{{\bar{p}}^{1}-{\bar{p}}^{2}}{var^{1}/n^{1}+var^{2}/n^{2}}$$

where ${\bar{p}}^{1}$, *n*^1 ^and *var*^1 ^are the mean value of features, the number of examples and variance of one class, ${\bar{p}}^{2}$, *n*^2 ^and *var*^2 ^have the similar meaning for the other class.

The meaning of t-statistic value is intuitive, which measures the weighted distance between the centroid of one class and the other class on the feature *p*. It is usually believed that the value of *t*(*p*|*Y*) largely represents the discriminative power of the giving feature, higher absolute score means greater discriminative power. A feature is usually regarded as irrelevant when its t-statistic value is trivial. The t-statistic measure has demonstrated as an effective feature selection method and widely used in the field of bioinformatics [20].

In total, we define the merit function of REMI as:

(4) $$J_{REMI}\left(X_{p}|S,Y\right)=\text{abs}\left(t\left(X_{p}|Y\right)\right)-\frac{1}{p-1}\overset{p-1}{\underset{i=1}{\sum }}REMI\left(X_{p};X_{i}|Y\right)$$

which denotes the merit score of candidate feature *X_p _*given the selected features *S *(*S *= *X*_1:*p*−1_) and the label *Y*. Furthermore, we propose a novel algorithm named RESI (Redundant fEature Selection depending on Instance), which combines REMI with the sequential forward search strategy. The detail algorithm is described as in Algorithm 1.

**Algorithm 1 **The RESI Algorithm

**Input: **Feature set *X *= [*X*_1_, *X*_2_, . . . , *X_m_*]

   Target label *Y*

**Output: **Selected feature subset *S*

1: *F *⇐ *X*;

2: *S *⇐ Ø;

3: **for all **feature *X_i _*in *F ***do**

4:     **if **abs(*t*(*X_i_*|*Y*)) <*∈ ***then**

5:         *F *⇐ *F \ X_i_*;

6:     end if

7: end for

8: $X_{p}⇐\text{arg}\;{\text{max}}_{X_{p}\in F}\text{abs}\left(t\left(X_{p}|Y\right)\right)$;

9: *S *⇐ *S *∪ *X_p_*;

10: *F *⇐ *F \ X_p_*;

11: **while **pre-defined stopping criteria is not satified **do**

12:     $X_{p}⇐\text{arg}\;{\text{max}}_{X_{p}\in F}J_{REMI}\left(X_{p}|S,Y\right)$;

13:     *S *⇐ *S *∪ *X_p_*;

14:     *F *⇐ *F \ X_p_*;

15: **end while**

The RESI algorithm works in two stages, including irrelevant features removing and redundant feature elimination. Firstly, the t-statistic value is computed for each feature. The irrelevant features, whose absolute t-statistic values are trival *i.e*. abs(*t*(*X_i_*|*Y*)) <*∈*, are removed from the candidate set. Then, the most discriminative features with the highest abs(*t*(*X_p_*|*Y*)) are selected. In the stage of redundant feature elimination, RESI starts with the current selected subset, and adds one important feature at a time. Given a selected feature subset of *p*−1 features *S *, a new feature *X_p _*is chosen from the rest of the feature subset *X \ S *by evaluating the merit function *J_REMI _*(*X_p_*|*S , Y*). It will be terminated when pre-defined stopping criteria is satisfied. For example, the number of selected features is larger than a threshold or the score of *J_REMI _*(*X_p_*|*S, Y*) is trivial.

In our experiments, the parameter *∈ *= 0.1, the number of neighborhood instances *k *= 3, the threshold of clear-discerned instance *µ *= 0.66.

### Related works on redundant feature detection

As we mentioned in Section Introduction, the predictive power and the inner redundancy of the selected feature subset are two key points in feature selection methods. In recent years, researchers have proposed various feature selection methods [21-23], most of which are explained from the two aspects. Here, we give a short review on related feature selection methods using forward feature selection scheme. These methods are briefly described in Table 1.

**Table 1:** Related feature selection methods

Methods	Predictive power	Redundancy measure	Merit function of candidate feature	Ref
CFS	*I*(*X_i_; Y*)	*I*(*X_p_; X_i_*)	$\text{Δ}\left(\frac{\sum_{i=1}^{p}I\left(X_{i};Y\right)}{\sqrt{p+\sum_{i=1}^{p}\sum_{j=1}^{p}I\left(X_{i};X_{j}\right)}}\right)$	[12]
MIFS	*I*(*X_p_; Y*)	*I*(*X_p_; X_i_*)	$I\left(X_{p};Y\right)-\beta \sum_{i=1}^{p-1}I\left(X_{p};X_{i}\right)$	[13]
mRMR	*I*(*X_p_; Y*)	*I*(*X_p_; X_i_*)	$I\left(X_{p};Y\right)-\frac{1}{p-1}\sum_{i=1}^{p-1}I\left(X_{p};X_{i}\right)$	[16]
MIFS-U	*I*(*X_p_; Y*)	$\frac{I\left(X_{i};Y\right)}{H\left(X_{i}\right)}I\left(X_{p};X_{i}\right)$	$I\left(X_{p};Y\right)-\beta \sum_{i=1}^{p-1}\left[\frac{I\left(X_{i};Y\right)}{H\left(X_{i}\right)}I\left(X_{p};X_{i}\right)\right]$	[14]
mIMR, JMI	*I*(*X_p_; Y*)	*I*(*X_i_; X_p_; Y*)	$I\left(X_{p};Y\right)-\frac{1}{p-1}\sum_{i=1}^{p-1}\left[I\left(X_{i};X_{p}\right)-I\left(X_{i};X_{p}|Y\right)\right]$	[17],[24]
CMIM, IF	*I*(*X_p_; Y*)	*I*(*X_i_; X_p_; Y*)	*I*(*X_p_; Y*) − max_*i *∈[1:*p*−1]_[*I*(*X_p_; X_i_*) − *I*(*X_p_; X_i_*|*Y*)]	[18],[25]

Hall proposed the Correlation-based Feature Selection (CFS) method [12], where some correlation measures are used to evaluate the goodness of a subset by considering the individual predictive ability of each feature and the degree of correlation between them. The symmetrical uncertainty (a normalized version of mutual information), for discrete data, and the standard linear correlation, for continuous data, are used by Hall to measure *I*(*X_i_; Y*) and *I*(*X_i_; X_j_*).

Ding and Peng proposed the minimum Redundancy-Maximum Relevance (mRMR) method in 2005 [16], which requires that selected discriminative features are maximally dissimilar to each other. mRMR is almost as the same as the MIFS method [13], except that the parameter *β *is set as $\frac{1}{p-1}$ in mRMR. Both MIFS and mRMR use mutual information to measure *I*(*X_i_; Y*) and *I*(*X_i_; X _j_*). Kwak and Choi proposed an improvement to MIFS, called MIFS-U [14], which uses a re-weighted mutual information $\frac{I\left(X_{i};Y\right)}{H\left(X_{i}\right)}I\left(X_{p};X_{i}\right)$ to measure the feature redundancy. Without explicit claim, *I*(·;· ) is measured by the mutual information in the rest of paper.

Bontempi and Meyer proposed a causal filter selection method, called min-Interaction Max-Relevance (mIMR) [17]. Bontempi and Meyer try to maximize the mutual information between *X*_1:*p *_and *Y *directly. Due to the number of the subset of *X*_1:*p *_is *O*(2*^p^*), it is impractical to compute *I*(*X*_1:*p*_; *Y*) in a precise way. Only low-order interactions are considered in the approximate solution given by mIMR. And in the final solution, the merit function of mIMR can also be divided into two parts: predictive power and feature set redundancy. Obviously, the difference between mRMR and mIMR is the two-way mutual information *I*(*X_i_; X_p_*) is replaced by the three-way mutual information *I*(*X_i_; X_p_; Y*), and *I*(*X_i_; X_p_; Y*) = *I*(*X_p_; X_i_*) − *I*(*X_p_; X_i_*|*Y*).

Fleuret used the Conditional Mutual Information Maximization (CMIM) to select feature [18], which examines the information between a feature and the target, conditioned on each current feature. It is clear that CMIM is very similar with mIMR. The only difference is the sum function is replaced by the maximum function to measure the feature redundancy.

Yang and Moody proposed using Joint Mutual Information (JMI) to select feature [24], which tries to maximize joint mutual information $\sum_{i=1}^{p-1}I\left(X_{p},X_{i};Y\right)$. This is the information between the targets and a joint random variable, defined by pairing the candidate *X_p _*with each current selected feature. But after deduction, the merit function of JMI is exactly equivalent to that of mIMR.

Vidal-Naquet and Ullman proposed another criterion used for computer vision, which is refereed as Informative Fragments (IF) [25]. The authors motivate the criterion min_*i*∈[1:*p*−1]_[*I*(*X_p_, X_i_; Y*) − *I*(*X_i_; Y*)] by noting that it measures the predictive ability gain of combining a new feature *X_p _*with each existing feature *X_i_*, over simply using *X_i _*by itself. The *X_i _*with the least "gain" from being paired with *X_p _*is taken as the score for *X_p_*. Interestingly, using the chain rule *I*(*X_p_, X_i_; Y*) = *I*(*X_i_; Y*) + *I*(*X_p_; Y*|*X_i_*), therefore IF is equivalent to CMIM.

From the short review, we can easily find the common point of these methods. Although the motivations are various, the merit functions of all these methods are divided into two parts: predictive power and feature redundancy. And due to the practical limitation, one certain pair-wise similarity measure is adopted to compute the predictive power and feature redundancy. However, the traditional pair-wise similarity measures, *i.e*. mutual information or conditional mutual information, only consider the numerical values of given variables, but not the the similarity of discriminative ability between them. Therefore, feature redundancy can not be measured correctly in existing methods.

### Data sets

There are fourteen data sets used in our study, which are listed in Table 2. The data set of Arcene-NIPS2003 is gathered from the NIPS'03 feature selection competition [26], Breast-Duke is reported by West [27], all other data sets are downloaded from the Kent Ridge Bio-medical Dataset [28]. All these data sets have relative big feature/instance ratio, and the feature numbers are no less than 2,000. For the missing values in some existing data sets, they are replaced by the corresponding means. For the data set of OvarianQStar, only the first 373,401 features are used.

**Table 2:** Experimental data sets

Data sets	Instances	Class ratio	Features
Arcene-NIPS2003	900	398/502	10,000
Breast	97	46/51	24,481
Breast-Duke	44	21/23	7,129
CNS	60	21/39	7,129
Colon	62	22/40	2,000
DLBCL-Stanford	47	23/24	4,026
DLBCL-Tumor	77	19/58	6,817
DLBCL-NIH	240	102/138	7,399
Leukemia	72	25/47	7,129
Lung	181	31/150	12,533
Lung-Michigan	96	10/86	7,129
Lung-Ontario	39	15/24	2,880
OvarianPBSII	253	91/162	15,154
OvarianQStar	216	95/121	373,401

### Experimental settings

We use the stratified 10-fold cross-validation procedure, where each data set is split into ten subsets of equal size. Each subset is used as test set once, and the corresponding left subsets are combined together and used as training set. Within each cross-validation fold, the gene expression data are standardized. The expressions of the training set are transformed to zero mean and unit standard deviation across samples, and the test set are transformed according to the means and standard deviations of the corresponding training set. The Irani's MDL method is applied when discretization is required [29]. The 10-fold cross-validation is repeated 10 times, which is also denoted as the 10 × 10 cross-validation measuring procedure.

We should note that the 10 × 10 cross-validation measuring procedure is more reliable than the randomized re-sampling testing strategy and the leave-one-out cross-validation due to the correlations between the test and training sets, some detailed discussions can be found at [30] Even in the small sample problem like gene expression data, 10 × 10 cross-validation is still one of the most reliable measuring way [31].

The final classification performance is recorded by the Balanced ACCuracy (BACC), which is defined as follows.

(5) $$\begin{array}{cc} BAAC & =\frac{1}{2}\left(sensitivity+specificity\right)\\ & =\frac{1}{2}\left(\frac{TP}{TP+FN}+\frac{TN}{TN+FP}\right) \end{array}$$

where TP, TN, FP, and FN, stand for the number of true positive, true negative, false positive, and false negative samples, respectively. Without explicit clarification, all the scores are averaged on 10 × 10 cross-validation.

To make conclusions sound, six widely used classifiers are used, including Support Vector Machine (SVM) with linear kernel and *c *= 1, non-linear support vector machine using Sequential Minimal Optimization (SMO) and polynomial kernel, *k *Nearest Neighbor (*k*NN) with *k *= 3, Logistic Regression (LR), Naïve Bayes classifier (NB) and decision tree with J48 algorithm (J48). All these classifiers are trained on the training set to predict the label of the test samples on the same cross-validation partition.

The algorithms are implemented in JAVA language based on WEKA [32], and carried out on a DELL PC workstation with 24 × X5680 3.33GHz CPU and 64G RAM.

## Results and discussion

**Figure 1:**
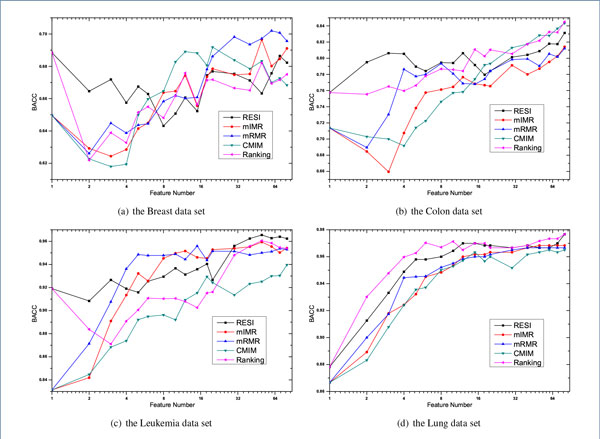
Comparative BACC results on four selected data sets by using *k*NN

**Figure 2:**
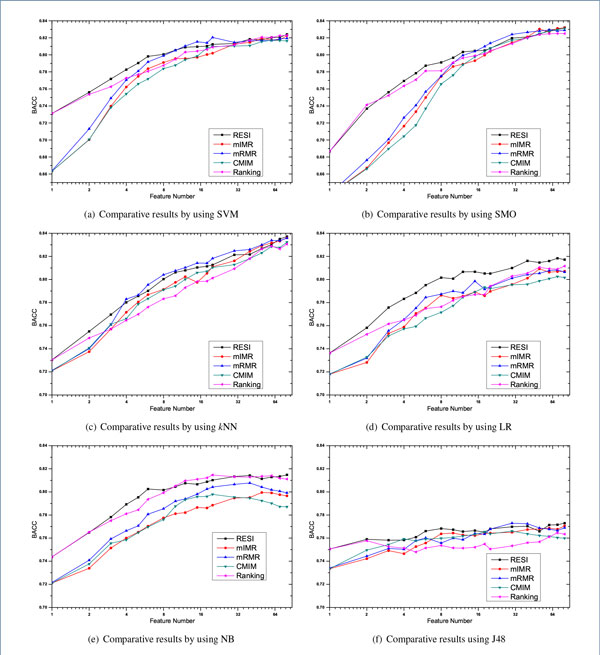
Averaged comparative BACC results on fourteen data sets by using different classifiers

In order to examine the performance of our proposed method, three state-of-the-art feature selection methods, mIMR, mRMR and CMIM, are used to compare with RESI. Additional, a feature Ranking method using absolute t-statistic score is also used as baseline. The parameter of selected feature number has great influence on the performance comparison. We vary the selected dimension from 1 to 80, which are 1, 2, 3, 4, 5, 6, 8, 10, 12, 15, 18, 20, 30, 40, 50, 60, 70 and 80 in detail. Higher dimension is not included because the number of relevant genes (whose *I*(*X_i_; Y*) > 0) is limited on some data sets. On each data set and each selected feature dimension, six widely used classifiers *i.e*. SVM, SMO, LG, *k*NN, NB and J48 are applied to examine the performances with the procedure of 10 × 10 cross-validation. The detailed comparative BACC results with *k*NN classifier are plotted in Figure 1. Due to the length limitation of the paper, only results on four representative data sets, including the data set of Breast, Colon, Leukemia and Lung, are included in Figure 1, which are widely used by previous researchers [16,17]. The comparative BACC results averaged on all fourteen data sets are plotted in Figure 2. Note, the abscissas of Figure 1 and Figure 2 use the Log coordinate and the Log base is 2.

From Figure 1 and Figure 2, it's clear there is no method has overwhelming performance over all classifiers, although the plotted lines in Figure 2 fluctuate much more gently than the drawings on individual data set. However, our RESI is relatively the best one among all the feature selection methods. This is especially obvious by using the classifier of LR, and we will demonstrate the point by t-test later.

The performances of mIMR, mRMR and CMIM are often tied, and mRMR is the best one in most cases. We suggest it's because all these methods use mutual information to represent the predictive power, and the traditional mutual information of mRMR is the most robust one to estimate feature redundancy in our experiments.

It's also interesting to find that the performances of RESI and Ranking are obviously better than that of others, when the selected dimension is small, *i.e*. below 5. This is true with almost all classifiers. We believe the superiority is caused by the difference of two metrics, mutual information and the absolute t-statistic, used to represent the predictive power. The absolute t-statistic measures the weighted distance between the centroid of two classes, which is directly related to the discriminative power of the giving feature. On the contrary, mutual information represents the general information between two variables. So from our experimental results, the absolute t-statistic performs better than mutual information to represent the predictive power.

Investigating the difference between RESI and Ranking, we find that their performances are similar when the selected dimension is small. This is because the absolute t-statistic is used to represent the predictive power by both of them. And when only a few features are selected, the influence of feature redundancy is not obvious. But as the selected dimension grows, the impact of feature redundancy becomes more and more critical to the BACC performance. Eliminating redundant features is meaningful when the feature dimension is not small, which is coincided with Figure 2, where RESI is much better than Ranking when the dimension grows.

Paired two-side t-test is also used to examine the differences between those methods. The corresponding t-test results are showed in Table 3. Each cell (W/T/L) in Table 3 summarizes over all data sets and classifiers the wins/ties/losses in BACC (at the significance level of 0.05) comparing various feature selection methods each other. The last column of Table 3 gives the overall W/T/L values summarized on corresponding compared methods. From Table 3, it's clear that RESI is the best feature selection method.

**Table 3:** Comparative t-test results (wins/ties/losses) summarized on data sets and classifiers

	mIMR	mRMR	CMIM	Ranking	Total
RESI	40-33-11	36-37-11	41-33-10	31-36-17	148-139-49
mIMR	-	9-42-33	31-28-25	20-30-34	71-133-132
mRMR		-	36-36-12	23-32-29	103-147-86
CMIM			-	18-27-39	65-124-147
Ranking				-	119-125-92

## Conclusions

Redundant feature selection is an important topic in the field of bioinformatics. Here, we propose a novel redundant feature subset measure REMI by comparing feature predictive powers directly, which is recorded by its instance distribution explicitly including clear-discerned instances and blur-discerned instances. Furthermore, a novel feature selection method RESI based on REMI was proposed. Experimental results on benchmark microarray data sets demonstrate that RESI performs better than the state-of-the-art algorithms like mRMR on fourteen benchmark data sets.

Future works include improving its efficiency and applying it to more scientific fields.

## Competing interests

The authors declare that they have no competing interests.

## Authors' contributions

All authors read and approved the final manuscript. GZL proposed the algorithm and wrote this paper. XQZ jointly proposed the paper idea, collected the data, implemented the computation on the datasets and checked the manuscript.

## Declarations

Funding for open access charge: Natural Science Foundation of China.

This article has been published as part of *BMC Medical Genomics *Volume 7 Supplement 2, 2014: IEEE International Conference on Bioinformatics and Biomedicine (BIBM 2013): Bioinformatics in Medical Genomics. The full contents of the supplement are available online at http://www.biomedcentral.com/bmcmedgenomics/supplements/7/S2.
